# Effect of Sitting Posture on Sit-Skiing Economy in Non-disabled Athletes

**DOI:** 10.3389/fspor.2020.00044

**Published:** 2020-04-28

**Authors:** Kimmo Lajunen, Walter Rapp, Juha P. Ahtiainen, Stefan J. Lindinger, Vesa Linnamo

**Affiliations:** ^1^Faculty of Sport and Health Sciences, University of Jyväskylä, Jyväskylä, Finland; ^2^Olympic Training Centre Freiburg, Freiburg, Germany; ^3^Department of Food and Nutrition and Sport Science, Center for Health and Performance, University of Gothenburg, Gothenburg, Sweden

**Keywords:** paralympics, classification, competition, oxygen consumption, trunk movement

## Abstract

This study focused on resolving the differences in economy between two common sit-skiing postures used by disabled athletes, suspected to be the most and least effective. Ten experienced non-disabled male cross-country skiers went through an incremental testing protocol with an ergometer simulating double poling in two sitting postures “kneeing” and “knee-high.” The protocol consisted of 3 × 4 min steady-state stages (13, 22, and 34% of maximal sprint power output). Subjects' respiratory gases and heart rate were measured and blood lactate concentrations were determined. In addition, pulling forces and motion capture recordings were collected. Oxygen consumption was 15.5% (*p* < 0.01) higher with “knee-high” compared to “kneeing” at stage three. At stage three cycle rate was 13.8% higher (*p* < 0.01) and impulse of force 13.0% (*p* < 0.05) and hip range of motion 46.6% lower (*p* < 0.01) with “knee-high” compared to “kneeing.” “Kneeing” was found to be considerably more economical than “knee-high” especially at 34% of maximum sprint power output. This might have been due to higher cycle rate, lower impulse of force and smaller hip range of motion with “knee-high” compared to “kneeing.” This indicates that sit-skiers should adopt, if possible, posture more resembling the “kneeing” than the “knee-high” posture. Combining such physiological and biomechanical measurements and to further develop them to integrated miniature wearable sensors could offer new possibilities for training and testing both in the laboratory and in the field conditions.

## Introduction

Cross-country skiing is one of the six sports in the winter Paralympic Games (International Paralympic Committee, 2020d) and sit-skiers form one of the three major sport classes in cross-country skiing (International Paralympic Committee, 2020b). In a sit-skiing event, each athlete sits on a sledge mounted on top of a pair of traditional cross-country skis and creates forward propulsion using the double poling (DP) technique (Gastaldi et al., 2012). DP in general refers to a skiing technique in which both poles are planted to the ground simultaneously and trunk flexion is synchronized with shoulder and elbow extension to create propulsive force (Smith et al., 1996). Due to the important role of the legs in DP of able-bodied skiers (Holmberg et al., 2005, 2006), the DP of sit-skiers is obviously different. For example, sit-skiers begin the poling phase with their hands above the level of their head (Gastaldi et al., 2012; Bernardi et al., 2013). Able-bodied skiers can utilize their full body mass to produce impulse to the poles (Holmberg et al., 2005), while disabled skiers are not able to do so.

To make sure that athletes can compete equitably with each other in sit-skiing they are classified based on their physical impairment and functional capability (International Paralympic Committee, 2020c). Locomotor Winter (LW) is a para-Alpine and para-Nordic sit-skiing classification defined by the International Paralympic Committee. Sit-skiers are allocated to five different classes: LW10, LW10.5, LW11, LW11.5, LW12. Class LW10 athletes have impairment affecting both their trunk and lower limbs. These athletes, while properly strapped over the legs to the test table, are unable to maintain a sitting position with their abdominal muscles or trunk extensors working against gravity without arm support. Class LW12 athletes' impairments are limited to their lower limbs (International Paralympic Committee, 2020c). Classes LW10–12 compete in the same category and a specific percentage-system is utilized to make competition equitable, which means that the competitor's actual finishing time is multiplied by the specific percentage to calculate their adjusted finishing time (International Paralympic Committee, 2020b). The current percentages for LW10–LW12 classes are 86, 90, 94, 97, and 100%, respectively (International Paralympic Committee, 2020a). These percentages have been determined based on the World Cup competition results from previous years.

**Figure 1:**
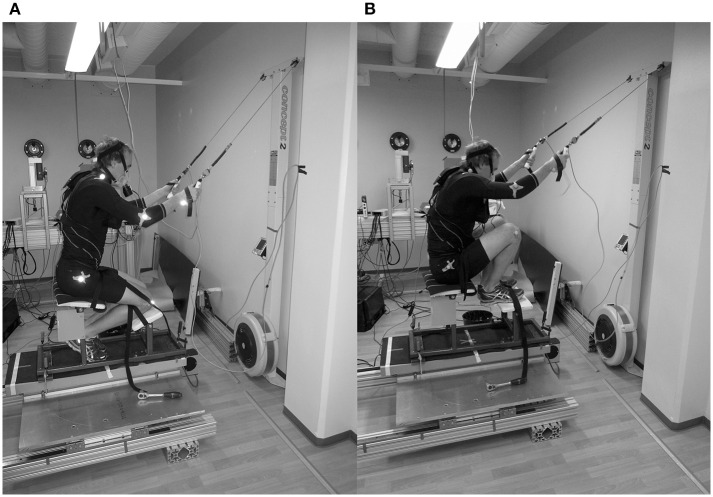
Illustration of the experimental setup during the **(A)** “kneeing” posture and **(B)** “knee-high” posture.

In Paralympic sit-skiing, two common sitting postures are observable primarily based on the level of impairment of the athletes. The rules do not designate athletes in a certain class to use a certain posture but the athletes try to obtain the position that would be most optimal with them. To achieve a stable sitting posture, athletes with high impairment (LW10 and LW10.5) use a posture where the knees are higher than the pelvis (“knee-high”) (see Figure 1). The other posture, “kneeing,” enables a more extensive hip range of motion (ROM) compared to the “knee-high” posture and is preferred by athletes with full trunk function. Gastaldi et al. (2012) showed that a skier using the “kneeing” posture has considerably more extensive trunk movement compared to a skier sitting in the “knee-high” posture. This is supported by our recent findings with able-bodied athletes that not only is the hip ROM smaller but also less power and lower maximal velocity can be obtained with the “knee-high” posture as compared to the “kneeing” posture (Rapp et al., 2013).

The importance of skiing economy for performance has been demonstrated in several studies in able-bodied athletes (Mahood et al., 2001; Mikkola et al., 2010; Ainegren et al., 2013). Mahood et al. (2001) observed a strong correlation between skiing performance and skiing economy. More recently, Ainegren et al. (2013) demonstrated that elite cross-country skiers had better skiing economy than recreational skiers and senior elite skiers were more economical compared to elite juniors. It has also been noted that there is a negative relationships between the velocity in a simulated sprint competition and oxygen consumption and blood lactate concentration in a 2 km constant velocity DP test (Mikkola et al., 2010).

Paralympic athletes differ based on their impairment level, functional capacity, and technique (International Paralympic Committee, 2020c). The classification process, however, may not take into account the additional disadvantage that some skiers face due to their sitting postures. To ensure that sit-skiers with various levels of impairments and different sitting postures can compete equally, it is essential to understand how sitting posture affects sit-skiing economy. In order to solely determine the difference in economy between different postures one posture should not be more favorable for some subjects than for the others. Disabled skiers have a wide variability in level of impairment and they become accustomed to one posture during their training. These issues could affect scientific evaluation of the two postures. Therefore, the aim of this study was to examine the differences in respiratory gases, blood lactate, force production and joint kinematics in non-disabled athletes between two common sit-skiing postures (“kneeing” and “knee-high”) observable in Paralympic sit-skiing competitions. This information could help coaches improve sitting postures for their athletes and possibly provide more scientific knowledge from which to base the classification system. It was hypothesized that due to larger trunk range of motion the “kneeing” position would be more effective and economical than “knee-high” position.

## Methods

### Experimental Approach to the Problem

An experimental study was designed to examine the effect of sitting posture on sit-skiing economy and associated biomechanical factors. The testing protocol consisted of 3 × 4 min incremental stages with both postures on a ski ergometer (Concept2, Morrisville, Vermont, USA) simulating DP. The resistance of the ergometer was set at six (scale 1–10) throughout the present study. To eliminate the effect of order, each stage was performed in both postures in a randomized fashion before proceeding to the next stage and the starting posture was randomized. Recovery periods between the stages were 2 min. Before the incremental protocol subjects warmed up for 5 min during which they carried out two short (5–10 s), near maximal sprints. After warm-up, subjects completed one maximal sprint (10–15 s) in the “kneeing” position in which the athletes were expected to achieve the highest maximal power output, which was then recorded. In order to compare the same absolute working loads powers outputs corresponding to 13, 22, and 34% of this maximum value were the stages used in the incremental protocol with “kneeing” and “knee-high” sitting positions. These two extreme positions were chosen based on our previous study where we examined four different sitting positions and observed the greatest differences in maximal velocity in these two positions (Rapp et al., 2013). During the stages, subjects were instructed to maintain the correct power output as accurately as possible. The current power output was displayed in real-time on the ergometer, which the authors carefully monitored and gave verbal feedback when necessary to maintain the target intensity.

### Subjects

Ten healthy male cross-country skiers (Age 22 ± 5 yrs., height 177 ± 6 cm, weight 71 ± 5 kg, reported VO_2_max 73 ± 5 ml•min^−1^•kg^−1^) volunteered to participate in the study. All participants had competed in cross-country skiing at the Finnish national level for at least 5 years. Before the start of the study, athletes were informed about the design of the study, with a special emphasis on possible risks and benefits, and they signed an informed consent document. In the case of one subject who was under 18 years old at the time of the study, parental consent was received. The study was performed according to the Declaration of Helsinki, and the Ethical Committee of the University of Jyväskylä approved the study.

### Procedures

A portable telemetry-based ergospirometer Cortex MetaMax 3B (Cortex Biophysik, Leipzig, Germany) was used for all respiratory gas measurements. This apparatus was connected (wirelessly) to MetaSoft 3.2 software on a computer. The variables of interest were oxygen consumption (VO_2_) relative to body weight, ventilation (VE) and respiratory exchange ratio (RER). Heart rate (HR) was measured using Polar Wearlink (Polar Electro Oy, Kempele, Finland) heart rate belt, which was also wirelessly connected to MetaSoft 3.2 software. Heart rate and respiratory variables were determined as average steady-state values during the last minute of each stage. Blood samples from the fingertip were taken within the first minute after each stage. From these samples blood lactate concentrations were determined using Lactate Pro (Carlton, Australia) portable blood lactate analyzer.

Pulling forces were measured using force –sensors (University of Jyväskylä, Finland), which were placed between the ergometer's handles and their strings. For kinematic analysis, six infrared cameras (Vicon Motion Systems Ltd., Oxford, UK) were used. In order to perform these recordings, small reflective markers were attached to the standardized positions in the subjects' knee, hip, shoulder, elbow, and wrist on the right side of the body. Illustrative examples of the experimental setup and the two sitting postures are shown in Figures 1A,B. Recordings of these biomechanical measurements were done synchronously during the second to last 30-s period of each stage using Vicon Nexus 1.8.3 software (Vicon Motion Systems Ltd., Oxford, UK). This software was also used to prepare the biomechanical data, which was ultimately analyzed with Ike Master 1.38 software (IKE Software Solutions, Salzburg, Austria). Biomechanical variables of interest were cycle rate, relative poling time, impulse of poling force and range of motion (ROM) of elbow, shoulder and hip joints. ROMs were analyzed in three dimensional space during poling phase (from the beginning till the end of the force production) as follows: Elbow ROM as a change in angle formed by lines between shoulder and elbow markers and elbow and wrist markers, shoulder ROM as a change in angle formed by lines between shoulder and elbow markers and shoulder and hip markers, and finally hip ROM as a change in angle formed by lines between shoulder and hip markers and hip and knee markers. All the biomechanical variables were analyzed as an average of nine consecutive cycles from the right side of the body.

### Statistical Analyses

The data was analyzed using SPSS software version 16.0 (SPSS Inc., Chicago, IL, USA). The results are expressed as mean ± SD. Sixty three of the total 66 variables were normally distributed and the normal Gaussian distribution of the data was verified by the Shapiro - Wilks test. A two-way, Posture (Bernardi et al., 2013) × Stage (Gastaldi et al., 2012), repeated measures ANOVA was performed to analyze the differences in the physiological and biomechanical variables between the two postures during the three stages. Furthermore, paired samples *T*-tests were run between the two postures on every stage and the Holm-Bonferroni method applied for the yielded *p*-values by multiplying all pairwise p-values with the number of comparisons conducted for each variable. The following variables were not normally distributed: VO_2_ at stage one in “knee-high” posture, blood lactate concentration at stage one in “kneeing” posture, and shoulder ROM at stage one in “kneeing” posture. Statistical differences between these three variables and their corresponding pairs were determined using non-parametric functions. Spearman's rank correlations were calculated between relative differences in oxygen consumption differences and relative differences in the biomechanical variables. In all statistical tests, differences were significant when *P* < 0.05.

## Results

### Power Outputs

The mean value for the power output in the maximum sprint was 292 ± 51 W. The power outputs for stages one, two, and three were 37 ± 6 W, 63 ± 11 W and 100 ± 18 W, corresponding to 13, 22, and 34%, of maximal power output, respectively.

### Differences in Physiological Variables

**Figure 2:**
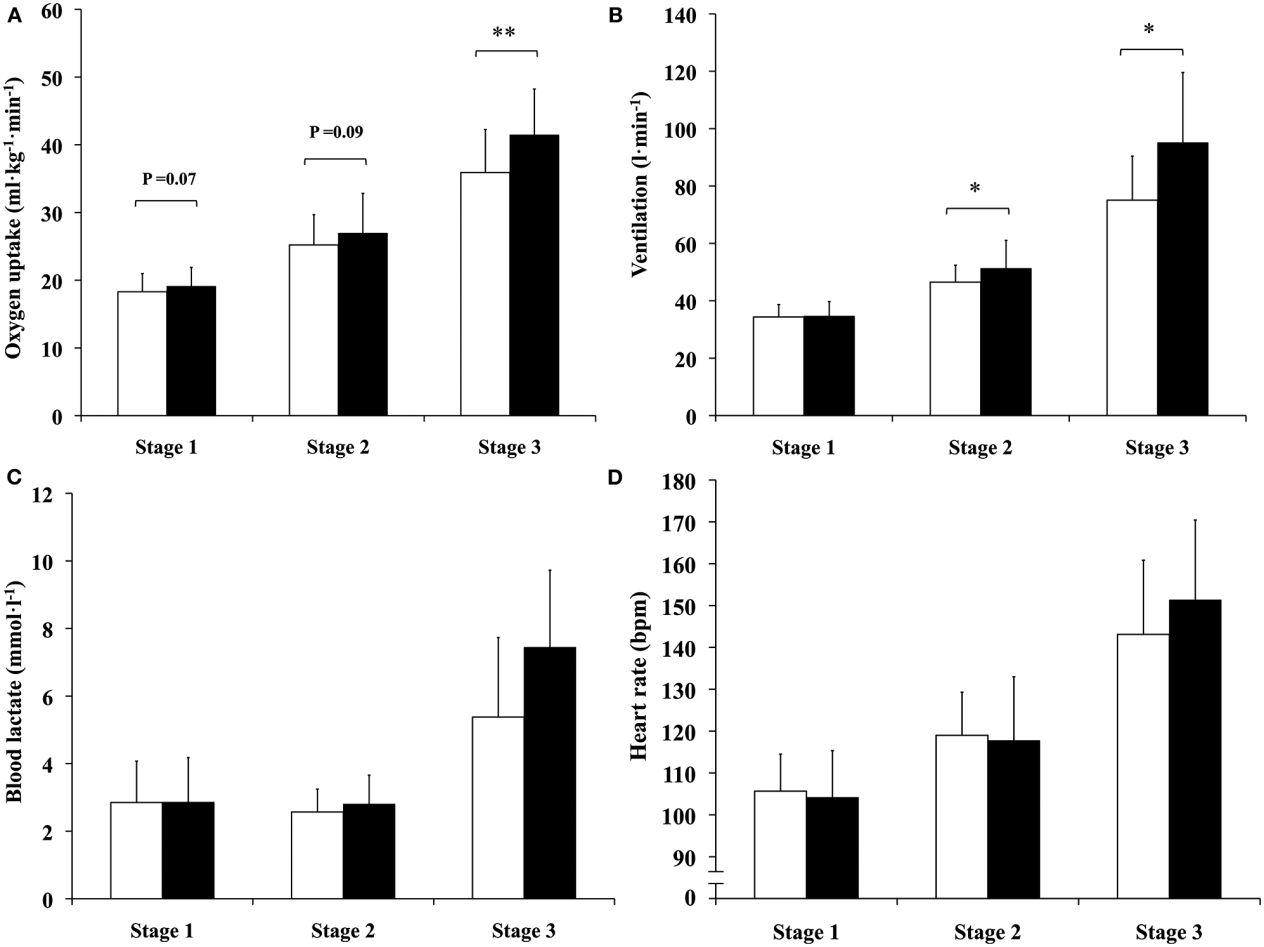
Differences in: **(A)** oxygen uptake, **(B)** ventilation, **(C)** blood lactate concentration, and **(D)** heart rate between “kneeing” (white columns) and “knee-high” (black columns) postures during stages one, two and three (13, 22 and 34 percent of maximum sprint power output, respectively). ^*^Statistically significant difference between “kneeing” and “knee-high” postures (^*^p < 0.05, ^**^p < 0.01).

Oxygen consumption (*P* < 0.01), ventilation (*P* < 0.05) and blood lactate concentration (*P* < 0.01) indicated significant differences between the two postures. Oxygen consumption was 15.5% (*P* < 0.01) higher with “knee-high” compared to “kneeing” posture at stage three (Figure 2A). Ventilation was 10.2 and 26.7% (both *P* < 0.05) higher with “knee-high” compared to “kneeing” posture at stages two and three, respectively (Figure 2B). No significant differences were observed in blood lactate concentration at any stage, despite a significant overall difference observed in ANOVA (Figure 2C). Heart rate (Figure 2D) and respiratory exchange ratio did not differ statistically during any load.

### Differences in Biomechanical Variables

**Figure 3:**
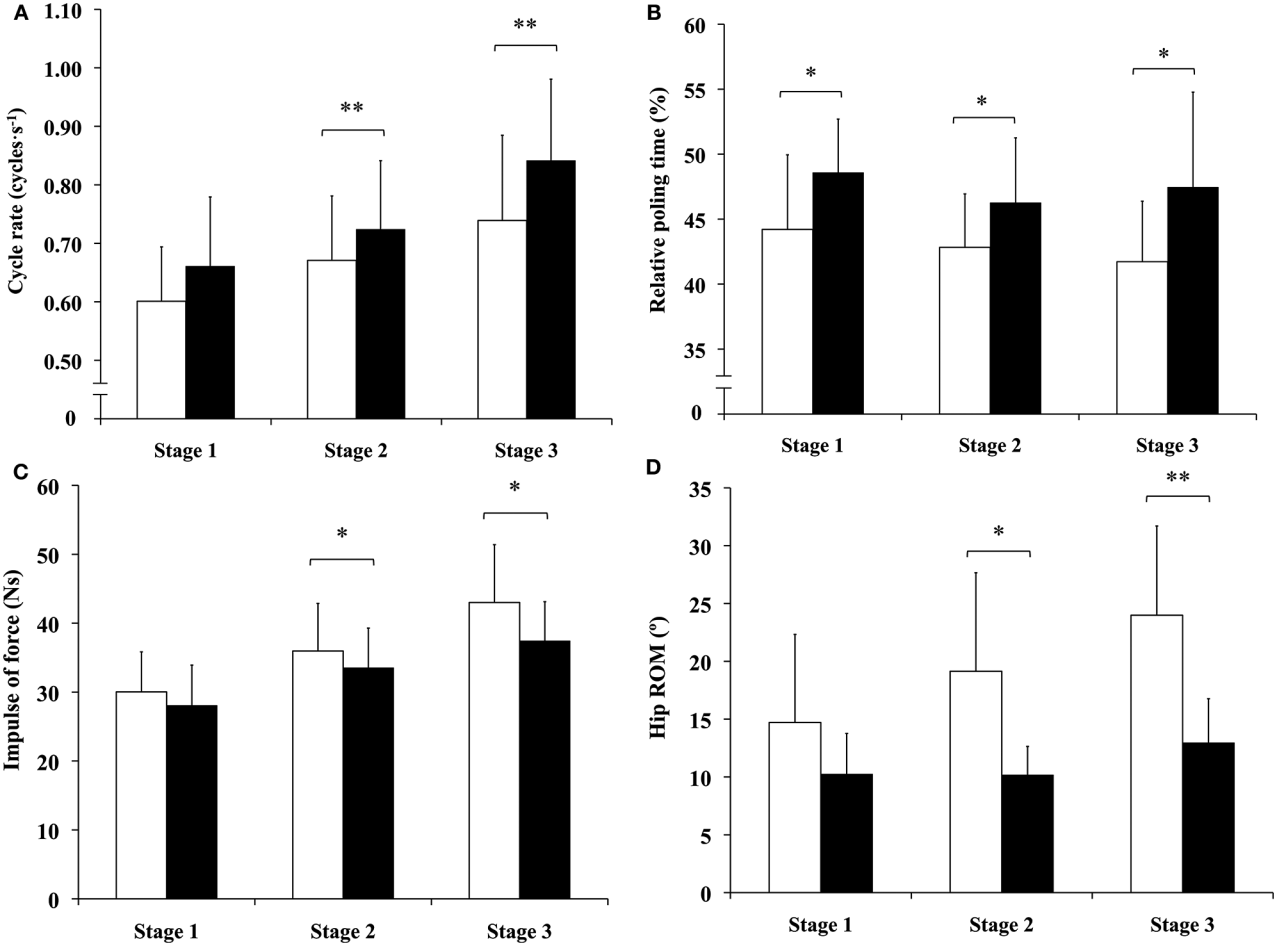
Differences in: **(A)** cycle rate, **(B)** relative poling time, **(C)** impulse of force, and **(D)** hip range of motion between “kneeing” (white columns) and “knee-high” (black columns) postures during stages one, two and three (13, 22, and 34 percent of maximum sprint power output, respectively). ^*^Statistically significant difference between “kneeing” and “knee-high” postures (^*^p < 0.05, ^**^p < 0.01).

Cycle rate, relative poling time, impulse of poling force and hip ROM demonstrated significant differences between the two postures (all *P* < 0.01). Cycle rate was 7.9 and 13.8% (both *P* < 0.01) higher with “knee-high” compared to “kneeing” posture at stages two and three, respectively (Figure 3A). Relative poling time was 9.9, 8.0, and 13.7% (all *P* < 0.05) higher with “knee-high” compared to “kneeing” posture at stages one, two and three, respectively (Figure 3B). Impulse of force was 6.8 and 13.0% (both *P* < 0.05) lower “knee-high” compared to “kneeing” posture at stages two and three, respectively (Figure 3C). Hip ROM was 46.8% (*P* < 0.05) and 46.6% (*P* < 0.01) smaller with “knee-high” compared to “kneeing” posture at stages two and three, respectively (Figure 3D).

### Correlations Between Differences in Economy and Biomechanical Variables

**Figure 4:**
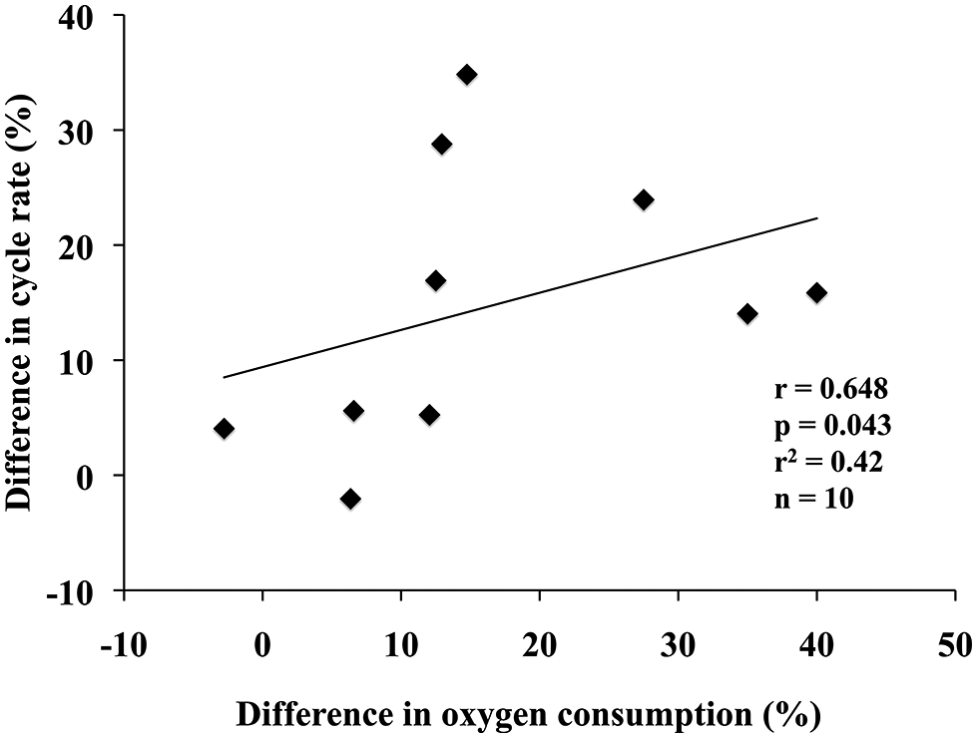
Positive correlation between relative differences of “kneeing” and “knee-high” postures in oxygen consumption and cycle rate during stage three (34 percent of maximum sprint power output).

Statistically significant correlations between posture-dependent differences in oxygen consumption and biomechanical variables were observed during stage three. Differences in cycle rate correlated positively to differences in oxygen consumption (*r* = 0.648, *P* = 0.043, *r*^2^ = 0.42) (Figure 4). In addition, differences in impulse of force (*r* = −0.636, *P* = 0.048, *r*^2^ = 0.40) and hip ROM (*r* = −0.667, *P* = 0.050, *r*^2^ = 0.44) correlated negatively to differences in oxygen consumption.

## Discussion

The main finding of present study was that oxygen consumption was 4.4–15.5% higher with the “knee-high” posture compared to the “kneeing” posture at matched power outputs. Hence, economy was better with the “kneeing” posture. Several other physiological variables supported this finding and posture-dependent differences were observed in a number of biomechanical variables. These findings confirm the results of Gastaldi et al. (2012) who also found that in Paralympic athletes the “kneeing” posture allows for a higher mechanical performance in Paralympic athletes.

The “kneeing” posture was found to be more economical compared to the “knee-high” posture. Most importantly, oxygen consumption at the same power output was higher with the “knee-high” posture. In addition, blood lactate concentration and ventilation were significantly higher with the “knee-high” posture. All of these inter-posture differences together provide strong evidence that the “kneeing” posture is more economical than the “knee-high” posture. This was expected based on our previous findings with able-bodied cross-country skiers (Rapp et al., 2013) in which the “kneeing” posture was shown to be considerably more effective than the “knee-high” posture.

In the present study, a number of biomechanical variables were measured to explain the differences in economy between postures. A higher cycle rate and lower impulse of force with the “knee-high” posture indicated that subjects compensated for a lower single cycle impulse by increasing poling frequency. Since relative poling time was also higher with the “knee-high” posture, it seems that subjects needed to shorten the recovery phase of the cycle to maintain the correct velocity. Our finding is in line with a previous study by Gastaldi et al. (2012) who reported that Paralympic athletes had considerably limited hip ROM with the “knee-high” posture. LW12 athletes, who most often use the “kneeing” position, have been shown to have greater trunk range of motion (ROM) compared to the lower classes both when skiing on snow (Gastaldi et al., 2012; Schillinger et al., 2016) and with an ergometer (Rosso et al., 2016). In the “knee-high” sit-skiing posture, trunk flexors cannot be activated extensively and they may operate at less effective muscle lengths compared to the “kneeing” posture. This could explain, at least in part, why the “knee-high” posture led to a higher cycle rate and lower impulse of force in the present study.

During stage three, the relative differences in cycle rate, impulse of force and hip ROM correlated significantly to the relative differences in VO_2_. The correlation between relative differences in cycle rate and relative differences in VO_2_ was positive. Correlations between relative differences in impulse of force and hip ROM and relative differences in VO_2_ were instead negative. These results suggest that a higher cycle rate, lower impulse of force and limited hip ROM with “knee-high” compared to “kneeing” posture are the most probable factors behind the higher VO_2_ with “knee-high” posture, although the significance levels and coefficients of determination of each factor individually were relatively low.

There were some limitations in this study. Two-minute rest periods between the stages may not have been sufficient to obtain full recovery even in our high-level athletes. However, by randomizing the order of the postures, any residual fatigue should have little influence on the study's main findings. The unfamiliarity of the sit-skiing movement may have caused technical difficulties for some subjects, especially during the maximal sprint. It seems unlikely though that these technical problems would have been more emphasized in one of the positions than in the other and thus the main purpose, which was to examine the effect of sitting position to performance, is probably not compromised. Nevertheless, although all efforts were made to simulate the real postures utilized by sit-skiing para-athletes, the laboratory conditions may not correspond to all conditions that athletes face in a racing situation. In a recent study by Rosso et al. (2017), it was, however, concluded that natural uphill (2.5°) skiing and ergometer skiing were comparable from a force production and muscle activation perspective. It remains to be studied how the sitting position affects cross-country skiing in downhills and curves where also balance control is very important.

Based on all the posture-dependent differences at stage three in this study, the disadvantage of using the “knee-high” instead of the “kneeing” posture could be over 15% at this level of intensity. In the percentage-system applied by the International Paralympic Committee, the compensation for an LW10 athlete compared to an LW12 athlete is 14% (International Paralympic Committee, 2020a). In this study, the difference between the two postures was more that this 14% compensation despite the fact that the physical condition of the athlete was the same in both postures. Therefore, an LW10 athlete forced to use the “knee-high” posture could suffer an additional disadvantage compared to an LW12 athlete using the “kneeing” posture due to their more severe impairment. Furthermore, athletes in the lower classes may also be able to use the “kneeing” posture and the compensation between classes LW10 and LW11 for example is only 8% (International Paralympic Committee, 2020a). The decision to use able-bodied athletes in this study was made due to high variety in level of impairment among disabled skiers that could confound the interpretation of the results. Moreover, the “kneeing” posture would be difficult or impossible for athletes in lower LW classes to perform. However, further research is needed to establish posture-dependent differences in sit-skiing economy among actual sit-skiers. For example, this could be possible by using athletes in higher LW classes only.

### Practical Applications

In previous studies concerning traditional cross-country skiing (Mahood et al., 2001; Mikkola et al., 2010; Ainegren et al., 2013) it has been concluded that good skiing economy is a crucial factor for succeeding in competitions. Given that observation and those of the present study, it can be recommended for coaches and sit-skiers to adopt a “kneeing” posture used in the present study. It is important to note, however, that not all sit-skiers are able to use the “kneeing” posture due to their impairment. In any case even a small use of trunk muscles during skiing may assist to train those important trunk muscles. Knee-high position seems to inhibit the use of the trunk even in non-disabled athletes so this position should be avoided if possible. Whether the current classification process and percentage-system accounts for this properly should be studied with disabled athletes.

## Data Availability Statement

Due to restrictions set by the ethics committee that approved this work, the datasets generated for this study will not be made publicly available. Requests to access these datasets should be directed to Vesa Linnamo, vesa.linnamo@jyu.fi.

## Ethics Statement

The studies involving human participants were reviewed and approved by Ethics Committee of the University of Jyväskylä. The patients/participants provided their written informed consent to participate in this study.

## Author Contributions

KL, WR, JA, and VL planned the study. KL and WR conducted the measurements. KL analyzed the data. All authors participated in the article writing.

## Conflict of Interest

The authors declare that the research was conducted in the absence of any commercial or financial relationships that could be construed as a potential conflict of interest.
